# Low-Temperature Metallization and Laser Trimming Process for Microwave Dielectric Ceramic Filters

**DOI:** 10.3390/ma14247519

**Published:** 2021-12-08

**Authors:** Jau-Jr Lin, Cheng-I Lin, Tune-Hune Kao, Meng-Chi Huang

**Affiliations:** 1 Department of Electrical Engineering, National Changhua University of Education, Changhua 500, Taiwan; bob135920000@gmail.com; 2 Mechanical and System Research Laboratories, Industrial Technology and Research Institute, Hsinchu 310, Taiwan; thkao@itri.org.tw (T.-H.K.); ach@itri.org.tw (M.-C.H.)

**Keywords:** microwave dielectric ceramics, dielectric filter, laser engraving, laser trimming, LTCC

## Abstract

This paper describes a low-temperature metallization and laser trimming process for microwave dielectric ceramic filters. The ceramic was metalized by electroless copper plating at a temperature lower than those of conventional low-temperature co-fired ceramic (LTCC) and direct bond copper (DBC) methods. Compared with filters made via traditional silver paste sintering, the metal in the holes of the microwave dielectric filters is uniform, smooth, and does not cause clogging nor become detached. Further, the batches of fabricated filters do not require individual inspection, reducing energy, labor, cost, and time requirements. A microwave dielectric filter was then manufactured from the prepared ceramic using a laser trimming machine with a line width and position error within ±50 μm; this demonstrates a more accurately controlled line width than that offered by screen printing. After using HFSS software simulations for preliminary experiments, the microwave dielectric filter was tuned to a target Wi-Fi band of 5.15–5.33 GHz; the return loss was <−10 dB, and the insertion loss was >−3 dB. To implement the real-world process, the laser parameters were optimized. Laser trimming has a higher success rate than traditional manual trimming, and the microwave dielectric filter manufactured here verified the feasibility of this process.

## 1. Introduction

Microwave dielectric ceramics are widely applied in many fields, such as mobile communications, wireless local networks, and satellite positioning and communications. Microwave dielectric ceramics with high dielectric constants and low loss tangents are suitable for devices such as filters, oscillators, and antennas [1,2,3]. The MgTiO_3_-CaTiO_3_ used in this study is a dielectric ceramic material that is widely used in microwave applications. The MgTiO_3_-CaTiO_3_ [4,5,6,7] has the desired properties for making compact and low-loss microwave devices, viz. a high dielectric constant (Dk), high quality factor/low loss (*Q* × *f*), and low-temperature coefficients of dielectric constant (TCK). Table 1 summarizes the properties of some MgTiO_3_-CaTiO_3_ materials.

**Figure 1:**
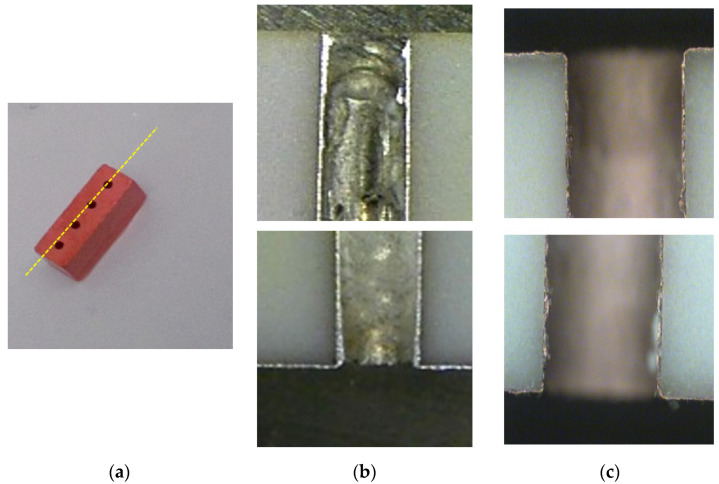
(**a**) A microwave dielectric ceramic filter; (**b**) Cross sections obtained with a conventional low-temperature co-fired ceramics (LTCC) process; (**c**) Cross sections obtained with the proposed low-temperature metallization process.

As the market for and volume of microwave dielectric ceramics grow continuously, processes that consume less power and time have been investigated globally [8,9,10]. For instance, novel low-temperature processes for microwave dielectric ceramic metallization have been proposed [11,12,13], including an electroless copper plating strategy previously developed by our group [13]. The temperature at which our previously fabricated metallization process operates is less than 50 °C; this avoids requirements of increasing and decreasing temperature, which saves both energy and time, respectively. Another benefit of this low-temperature process is that it results in metal with uniform and smooth thicknesses and other dimensions; this provides cross sections superior to those of conventional low-temperature co-fired ceramics (LTCCs), which are often clogged by incompletely attached metal (Figure 1).

A manufactured microwave dielectric ceramic filter with four through-holes is shown in Figure 1a; the through-holes were drilled using a laser. Figure 1b,c show the cross sections (along the dashed line shown in Figure 1a) of filters prepared with the conventional LTCC process and the low-temperature metallization process, respectively. Figure 1b,c show that the metal thickness and dimensions provided by the low-temperature metallization process are more uniform and smoother than those provided by the conventional LTCC process, which would avoid the metal clog problems associated with the traditional process. Furthermore, the batches of microwave dielectric filters do not need to be inspected separately, which saves energy, labor, cost, and time.

After the low-temperature metallization process, the frequency response band of the microwave dielectric ceramic filter was lower than the target specifications. Hence, a laser trimming process was introduced to modify the ceramic structure to shift the frequency response to the desired band. The low-temperature metallization and laser trimming process of the microwave dielectric ceramic filter are explained in Section 2. In Section 3, an electromagnetic 3D simulation software is used to understand the characteristic moving trend of the microwave dielectric ceramic filter after applying the laser trimming process. In Section 4, the laser trimming process is applied to real ceramic samples to determine the associations between the laser trimming parameter recipe and the digging depth. After a series of processes and testings, optimized laser trimming parameter recipes were obtained. In Section 5, the final process and measurement results are presented. This study is concluded in Section 6.

## 2. Low-Temperature Metallization Process and Filter Specification

**Figure 2:**
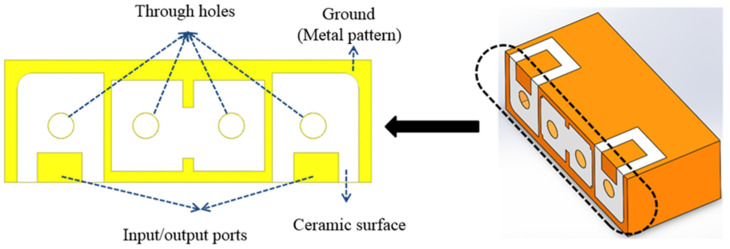
The microwave dielectric ceramic filter structure.

The structure of the microwave dielectric ceramic filter is shown in Figure 2.

**Figure 3:**
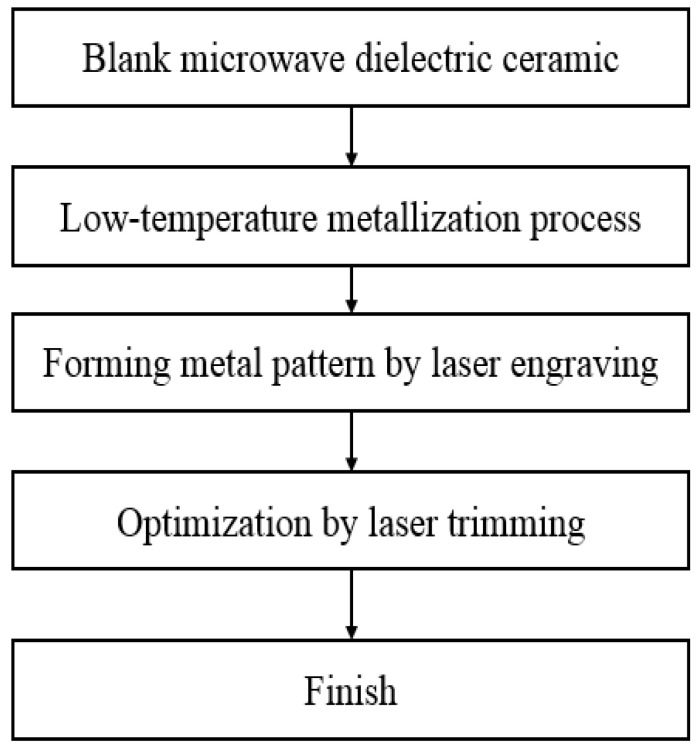
The complete low-temperature metallization and laser trimming processes.

There are four through-holes with metal on the inner walls and a designated metal pattern on the ceramic surface. The dimensions were approximately 8 × 3 × 4 mm^3^. The low-temperature metallization and laser trimming processes are shown in Figure 3.

**Figure 4:**
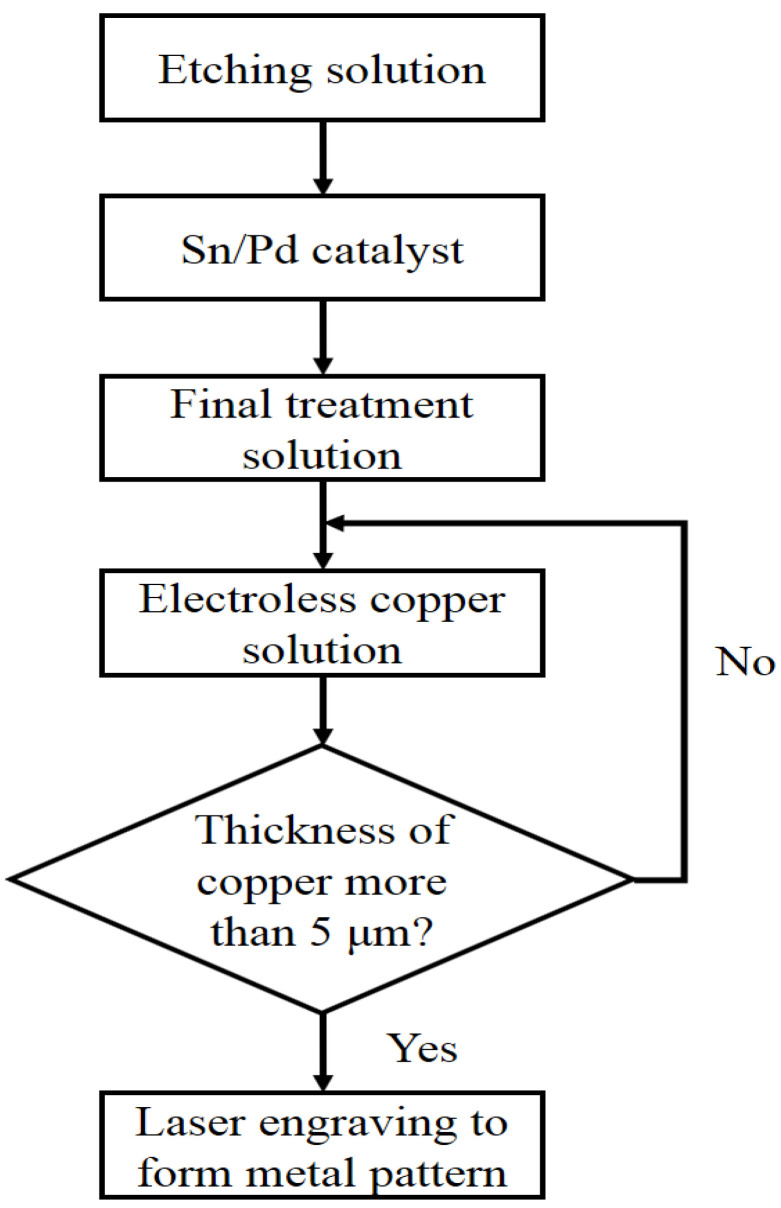
The detailed steps of the low-temperature metallization process.

First, a blank microwave dielectric ceramic with the desired dimensions was obtained. The microwave dielectric ceramic metallization technology involves processing the microwave dielectric ceramic surface and depositing a copper metal on it using electroless copper plating technology; the detailed steps are shown in Figure 4 [13]. 

To form micro-pores on the surfaces of the ceramics and their holes, the CaTiO_3_ component was dissolved and removed from the ceramic using an oxidative etching solution; (e.g., phosphoric acid and HCl). After five minutes in the etching solution, the solution color was relatively yellow, which suggests that the Ca/Ti particles dissolved in the solution. The etching solution roughens the ceramic surface, which helps metals to adhere to it. The ceramic was then kept in an activator for ten minutes; the activator contained Sn and Pd ions that adhered to the ceramic surface. The ceramic was kept in the cleaning agent for three minutes as a final treatment to remove any free Sn particles, leaving just the Sn ions on the surface. Finally, the ceramic was placed in a copper solution that was heated with a water bath for one hour, where the temperature was maintained between 40 and 50 °C to control the deposition rate at approximately 5–7 µm/h. If the temperature is lower than 40 °C, the process may be too slow; if the temperature is higher than 50 °C, the process may be too fast, which could cause uneven copper thickness and poor copper adhesion. Owing to this being a low-temperature technology and the process being rapid, this metallization method has high energy-saving benefits. The desired metal pattern was then formed by laser engraving. For this, the width and position of the circuit were accurately controlled with a minimum line width of less than 50 μm, and the position accuracy was controlled within ±50 μm. These first three steps were introduced in previous works [11,12,13].

The characteristics of the manufactured microwave dielectric ceramic filters differed from the specifications, as shown in Table 2.

The target passband is between 5.15 and 5.33 GHz; this targets a 5-GHz Wi-Fi application with channel numbers from 32 to 64. The input matching (S_11_) should be less than −10 dB and the in-band loss (S_21_) should be higher than −3 dB at 5.15 to 5.33 GHz. Note that f_L_ and f_H_ represent the lowest and highest frequencies, respectively, with S_11_ being less than −10 dB. The passband bandwidth (BW) is the difference between f_L_ and f_H_, and the image rejection (S_21_) should be less than −60 dB at ~5.5 GHz. This aims to suppress the possible interference from the 5.5 GHz Wi-Fi band. The filter structure is symmetric, which means S_11_ and S_22_ will be the same, as well as S_21_ and S_12_. Thus, only S_11_ and S_21_ are discussed.

## 3. Electromagnetic Simulations

Electromagnetic 3D simulation software (HFSS) was used to simulate and predict the results after laser trimming. In a previous study [13], the frequency band of the microwave dielectric ceramic filter was slightly shifted, and the in-band loss was higher than the specifications. Based on [14,15], the lengths of the four resonant cavities can be adjusted to achieve the appropriate frequency band and filtering effect. Therefore, the strategy is to process the through-holes. At the early stage of the simulations, all four holes were processed, but after the simulations, we discovered that processing the two middle holes would be enough to meet the filter specifications thereby saving time and cost in real laser trimming processes. Thus, the simulation process focuses on the two middle holes in this section. 

**Figure 5:**
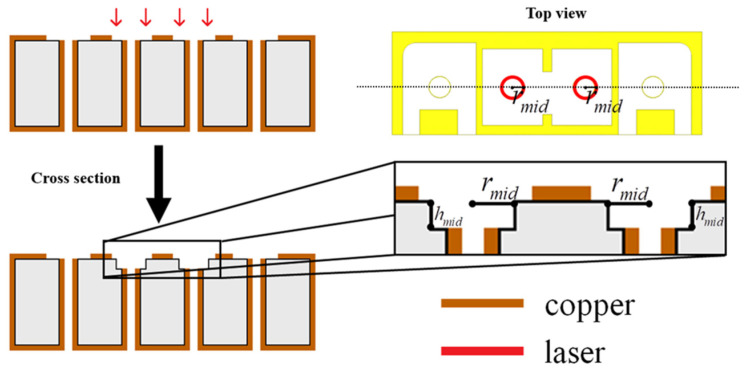
The top view and cross section of laser trimming processes.

Figure 5 shows the top view and cross section of the laser trimming process. The cross section is based on the dashed line in the top-view diagram in Figure 5. The laser trimming process modifies the digging depth (*h_mid_*) and digging radius (*r_mid_*) of the two middle holes to adjust the frequency band and improve the in-band matching and loss. HFSS simulations were used to determine the trends with various *h_mid_* and *r_mid_* values, as well as to suggest the final *h_mid_* and *r_mid_* parameters for the real-world laser trimming performed later. The hole radius used in this project was 0.35 mm with an approximate error of ±25 µm. Therefore, the hole digging radius was initially set to a minimum of 0.4 mm and evaluated at different digging depths; a hole digging radius of 0.5 mm was similarly investigated.

**Figure 6:**
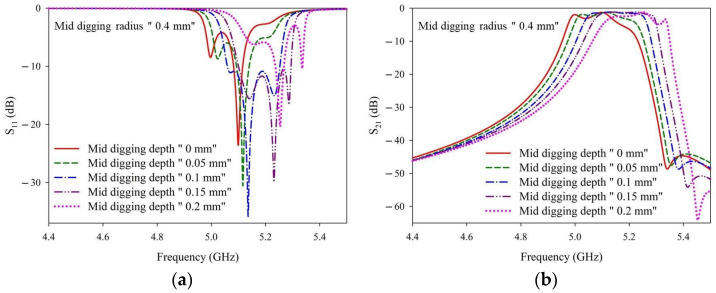
(**a**) S_11_ and (**b**) S_21_ simulation results with a fixed digging radius of 0.4 mm and various digging depths.

Figure 6 shows the S-parameters of these simulation results. The specific numbers of the corresponding curves are provided in Table A1. When the radius of the digging hole was fixed at 0.4 mm and the depth was increased from 0 mm to 0.15 mm in increments of 0.05 mm, the S11 value tended to be lower, and the frequency band shifted to a higher frequency. The optimal depth falls between 0.1 mm and 0.15 mm; however, as the depth was further increased to 0.2 mm, the frequency band continued to shift toward higher frequencies but the S_11_ values increased.

**Figure 7:**
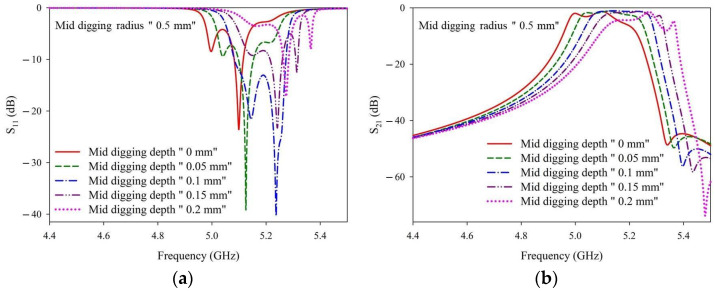
(**a**) S_11_ and (**b**) S_21_ simulation results with a fixed digging radius of 0.5 mm and various digging depths.

Figure 7 shows the S-parameter results with the digging radius fixed at 0.5 mm and various digging depths. The specific numbers of the corresponding curves are detailed in Table A2. The simulations run at digging radii of 0.4 and 0.5 mm showed similar trends, wherein the S11 value has the tendency to be lower, while the frequency band shifts to a higher frequency with a larger digging depth. When the fixed digging radius was 0.5 mm, the best performance was achieved with a digging depth of approximately 0.1 mm. The simulation results shown in Figure 6 and Figure 7 and Table A1 and Table A2 reveal that even with different digging radii, an appropriate digging depth can provide desired filter specifications. The digging depth in the subsequent simulations was set as 0.1–0.15 mm based on these obtained results.

**Figure 8:**
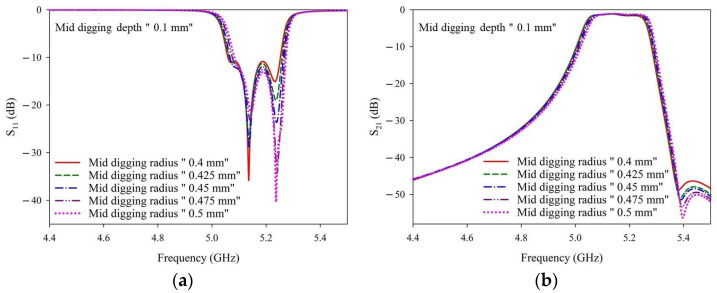
(**a**) S_11_ and (**b**) S_21_ simulation results with a fixed digging depth of 0.1 mm and various digging radii.

Figure 8 shows the simulated S-parameters with a fixed digging depth of 0.1 mm and various digging radii. The specific values of the corresponding curves are given in Table A3. At a fixed digging depth of 0.1 mm, the input matching (S_11_) will be slightly lower and the rejection (S_21_ at ~5.4 GHz) is slightly better with wider digging radius.

**Figure 9:**
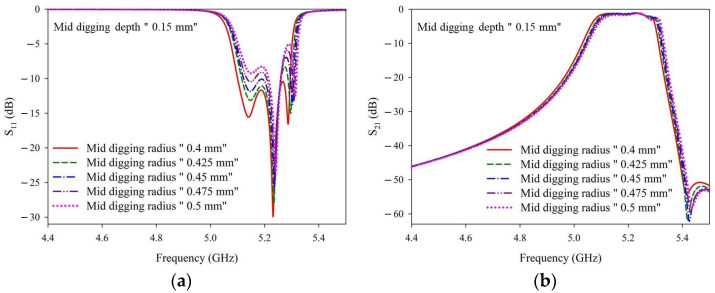
(**a**) S_11_ and (**b**) S_21_ simulation results with a fixed digging depth of 0.15 mm and various digging radii.

Figure 9 shows the simulated S-parameters obtained with a fixed digging depth of 0.15 mm and various digging radii. The detailed numbers of the corresponding curves are given in Table A4. With a fixed digging depth of 0.15 mm, the input matching (S_11_) was slightly higher/worse in the passband range. When considering the real-world laser trimming process, a YAG laser was applied. Owing to the heat accumulation around the operation areas, the more the YAG laser is utilized in the trimming process, the more difficult it is to control the digging depth and width after laser trimming. Therefore, a digging radius of 0.4 mm was used.

## 4. Laser Trimming Parameters, Operation Times, and Digging Depths

**Figure 10:**
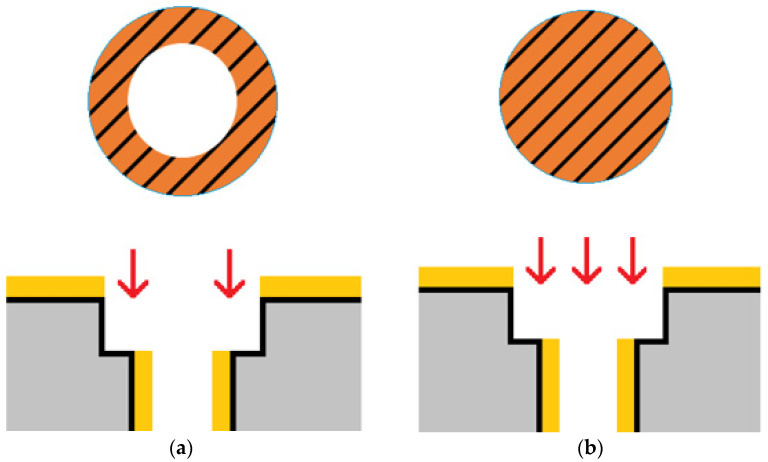
Two laser trimming strategies: (**a**) donut type and (**b**) circular type.

**Figure 11:**
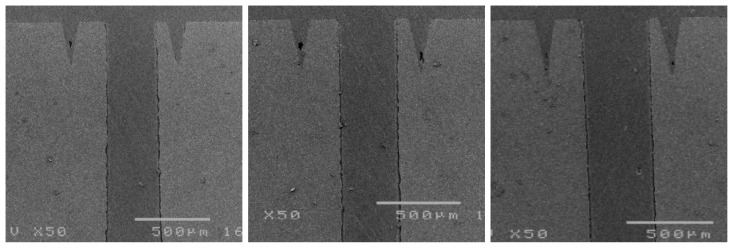
Cross sections of three samples unsuccessfully trimmed with a donut-type laser trimming strategy.

Simulations (Section 3) revealed that reducing the two middle holes shifts the frequency response to a higher frequency and smooths the frequency band. In this section, the laser trimming parameter recipe was investigated to meet the demands. Ceramic samples with and without metal surfaces were used for testing. After applying different laser trimming parameter recipes, the ceramic samples were cut and the digging depths were recorded. Four laser trimming parameters were evaluated: laser power (W), laser emitting frequency (kHz), processing speed (mm/s), and the number of repeated laser firings. These parameters were chosen because when a laser spot size is smaller than the designated trimming area radius, which is 350 µm in this study, the laser needs to repeatedly fire and move around the designated area to trim a uniform depth; this behavior can be investigated through the aforementioned parameters. Concerning notation, the parameter recipe of “5.5w_30k_300_re50” refers to a laser power of 5.5 W, a laser emitting frequency of 30 kHz, a processing speed of 300 mm/s, and 50 repetitions. In the early stage, the laser trimming zone was set as a donut type, as shown in Figure 10a. As mentioned in Section 3, the hole radius was 350 ± 25 µm. Consequently, a few smaller holes were not trimmed around the hole opening area, as shown in Figure 11. Figure 11 shows the cross-sectional images of the samples of three failed attempts using the donut-type laser trimming strategy. To avoid this type of failure, the laser trimming zone was changed to a circular type, as shown in Figure 10b.

**Figure 12:**
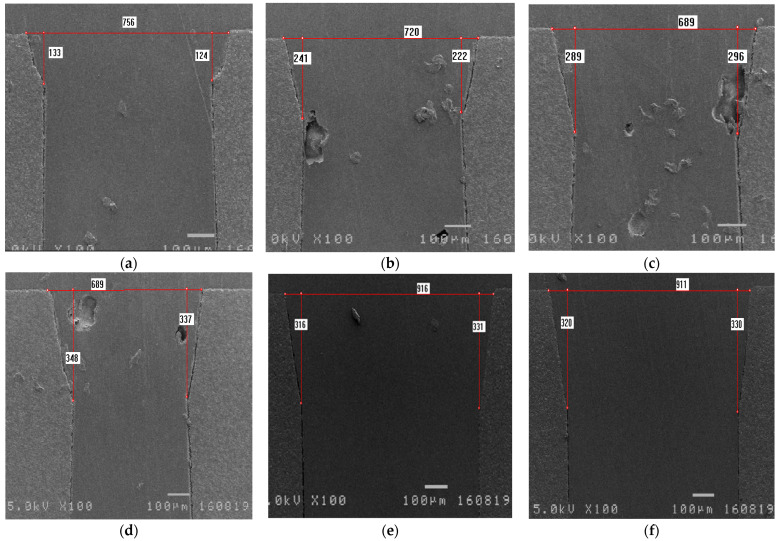
The cross sections of samples trimmed for different numbers of repeated laser firings with the 5.5w_30k_300_re50 recipe (no metal surface): (**a**) one; (**b**) two; (**c**) three; (**d**) four; (**e**) five; and (**f**) six repetitions.

Figure 12 shows the corresponding cross sections after different numbers of repeating laser firings with the 5.5w_30k_300_re50 recipe. Figure 12a–f show the cross sections of blank ceramic samples (i.e., those without metal surfaces) after one to six repetitions of laser firing, respectively; Table 3 summarizes the results shown in Figure 12. The results show that when the number of laser firings is more than three, the digging depths are approximately 325–361 µm because the laser goes out of focus while trimming deeper. For this process, the laser beam was set to focus on the ceramic top surface. As the number of repetitions increased, the hole edge became deeper. When the digging depth is greater than 300 µm, the laser goes out of focus and does not have enough power to dig deeper. 

Table 4 summarizes the cross-sectional results of the ceramic samples with a metal thickness of 15 µm on the surface. From Table 4, it can be seen again that when the number of laser firings is greater than three, the depths are approximately 300 µm. Further, these results for samples with metal surfaces (Table 4) are consistent with those for samples without metal surfaces (Table 3).

As the electromagnetic simulations suggested in Section 3, the digging depth was approximately 0.1–0.15 mm; therefore, the 5.5w_30k_300_re50 trimming parameter recipe provided depths that were too great for this study. Therefore, after trying many combinations of laser trimming parameters, the laser trimming parameter recipes of 4w_30k_300_re10, 4w_30k_300_re20, and 4w_30k_100_re10 were selected for further investigations. The corresponding cross-section depths are summarized in Table 5, Table 6 and Table 7, respectively, as are those of the blank ceramic samples without metal surface.

## 5. Final Manufacture Process and Measurement Results

**Figure 13:**
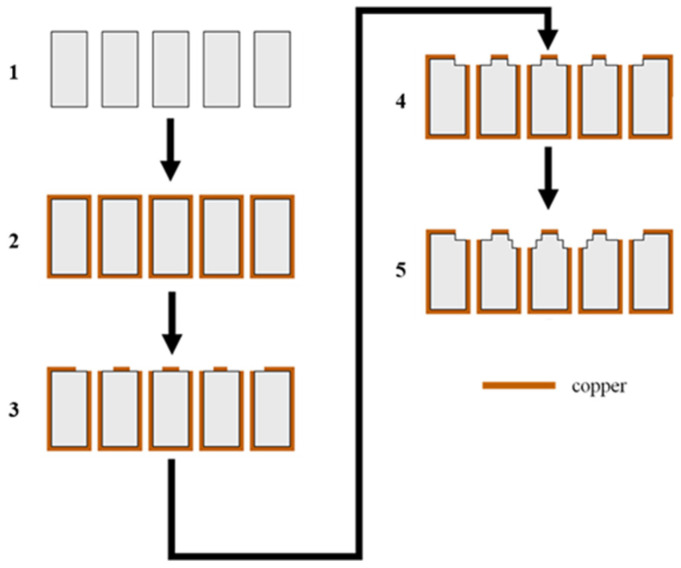
The final manufacturing process of the microwave dielectric ceramic filter.

The final manufacturing process of the microwave dielectric ceramic filter is illustrated in Figure 13.

In Step 1, holes were drilled into the blank ceramic, while Steps 2 and 3 employed our previously reported [13] low-temperature process metallization process with laser patterning (Steps 1–3 are explained in detail in [13]). In Step 4, the ceramic surface around all four holes was partially trimmed. In Step 5, a lower trimming depth was utilized on the two middle holes to obtain the optimized results.

**Figure 14:**
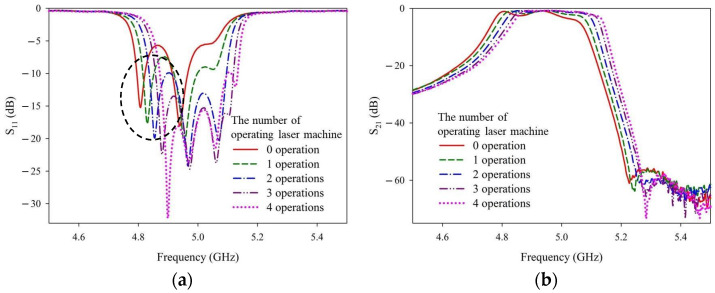
Measured (**a**) S_11_ and (**b**) S_21_ values after different numbers of operations with the 4w_30k_100_re10 recipe.

**Figure 15:**
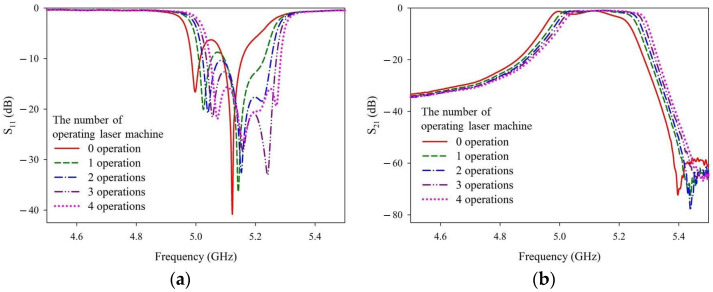
Measured (**a**) S_11_ and (**b**) S_21_ values after different numbers of operations with the 4w_30k_300_re20 recipe.

To obtain the approximate digging depth provided around the four holes in Step 4, the laser trimming parameter recipes of 4w_30k_300_re20 and 4w_30k_100_re10 were applied. After one operation (i.e., the number of repeated laser firings = 1), the ceramic filter sample was removed to measure and record the S-parameter characteristics. The ceramic filter sample was then returned and the process was repeated three more times (i.e., a total of four operations). Figure 14 and Figure 15 show the measurement results of the laser trimming parameter recipes 4w_30k_100_re10 and 4w_30k_300_re20, respectively.

The solid red curves represent ceramic filter samples without laser trimming. Figure 13 and Figure 14 show that the input matching (S_11_), in-band loss (S_21_), and band rejection (S_21_) shift toward higher frequencies with deeper digging depths (i.e., more operations) around the four holes. Upon comparing Figure 13 and Figure 14, several input matching (S_11_) curves in the passband lower end of recipe 4w_30k_100_re10 can be seen; these values are greater than −10 dB. However, these values are not as good as those of the 4w_30k_300_re20 recipe. Thus, recipe 4w_30k_300_re20 was selected for Step 4.

**Figure 16:**
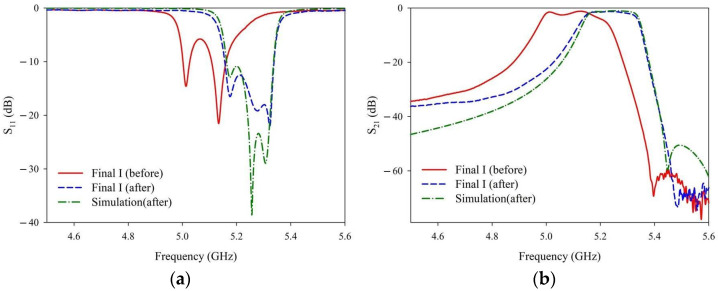
Measured (**a**) S_11_ (**b**) S_21_ values of the filter before laser trimming and after laser trimming (measurements and simulation results).

The final measurement results are presented in Figure 16 and Table 8. Figure 16 and Table 8 show the data before and after (measurements and simulation results) laser trimming. Step 4 of the final manufacturing process consists of two operation times using the 4w_30k_300_re20 recipe (Table 6), and Step 5 comprises another two operation times of the 4w_30k_300_re10 recipe (Table 5). As shown in Figure 16 and Table 8, the manufactured microwave dielectric ceramic filter meets design specifications after applying the proposed laser trimming recipes and manufacturing processes. 

**Figure 17:**
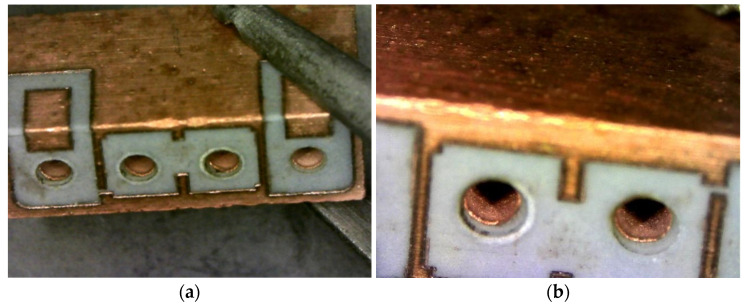
The manufactured microwave dielectric ceramic filter: (**a**) The laser trimming the opening parts of the through-holes; (**b**) Closer view of the two middle holes.

Figure 17 shows the photographs of the manufactured microwave dielectric ceramic filter. These photos clearly show that any metal around the hole openings was removed after laser trimming. At this stage, approximately ten microwave dielectric ceramic filters were manufactured in one round. The measurements were consistent among all ten, and met the target design criteria. This shows that the proposed recipes and processes are practical, effective, and can be applied to small-scale production. Comparisons between the conventional LTCC process and our proposed method are summarized in Table 9. The proposed process solves the metal clogging problem in the holes successfully. It also has less line width error and higher line position precision and can be applied to small-scale production.

## 6. Conclusions

In this study, microwave dielectric ceramics made of MgTiO_3_-CaTiO_3_ were metalized by electroless copper plating, and then patterned and optimized by a laser trimming process. Adopting this process can effectively save energy, production costs, and manufacturing time. The temperature employed for the entire microwave dielectric ceramic metallization process was below 50 °C. Thus, compared to other methods such as LTCC and DBC metallization, this technology can not only effectively reduce energy, but also greatly reduce the time and cost of heating and cooling. In addition, the proposed method solves the problem of clogging or incomplete adherence in the holes by applying a conventional silver paste sintering process. For laser engraving/trimming, the errors of the width and relative position of each line were within ±50 μm. Repeated laser trimming successfully adjusted the length of each resonant cavity to tune the device to the expected frequency band, and the digging depth was more accurately controlled than that observed for manual trimming. In addition to reducing the failure rate of product trimming, this strategy can also reduce labor costs and time. All measurements of the microwave dielectric ceramics fabricated using the proposed process met the desired design criteria. Moreover, approximately ten microwave dielectric ceramic filters can be manufactured in one round in the laboratory. The measurements among the ten filters were consistent, and met the criteria. This demonstrates that the proposed recipes and processes are practical and effective and can be applied to small-scale production.

## Figures and Tables

**Table 1:** The properties of selected MgTiO_3_-CaTiO_3_ materials.

Material	Dk	*Q* × *f* (GHz)	Sintering Temperature (°C)	TCK (ppm/°C)
MgTiO_3_ [4]	17	16,000 @ 7 GHz	800	−50
CaTiO_3_ [4]	170	3600 @ 7 GHz	800	800
0.95 MgTiO_3_-0.05 CaTiO_3_ + 2 wt%B_2_O_3_ [5]	21.2	62,000 @ 8 GHz	1400	4
0.95 MgTiO_3_-0.05 CaTiO_3_ + 3.5 wt%B_2_O_3_ [6]	20.3	68,000 @ 6 GHz	1210	−5
MgTiO_3_-CaTiO_3_ [7]	18–20	4000 @ 6 GHz	1360	−10 to 10

Abbreviations. Dk = dielectric constant, *Q* × *f* = quality factor at the corresponding frequency, TCK = temperature coefficient of dielectric constant.

**Table 2:** The microwave dielectric ceramic filter specifications.

Passband			Input and Output Matching (S_11_ and S_22_)	In-Band Loss (S_21_ and S_12_)	Image Rejection at ~5.5 GHz
f_L_ (GHz)	f_H_ (GHz)	BW (MHz)			
5.15	5.33	180	<−10 dB	>−3 dB	<−60 dB

**Table 3:** The measured depth values after different numbers of repeated laser firings with the 5.5w_30k_300_re50 (no metal surface) recipe.

Number of Repeated Laser Firings	Depth on the Left Edge (µm)	Depth on the Right Edge (µm)	Depth Difference (µm)	Depth Average (µm)
One	133	124	9	143.5
Two	241	222	19	231.5
Three	289	296	7	292.5
Four	348	337	11	342.5
Five	316	331	15	338.5
Six	320	330	10	325

**Table 4:** The measured depth values after different numbers of repeated laser firings with the 5.5w_30k_300_re50 (15-µm metal surface) recipe.

Number of Repeated Laser Firings	Depth on the Left Edge (µm)	Depth on the Right Edge (µm)	Depth Difference (µm)	Depth Average (µm)
One	170	174	4	172
Two	287	296	9	291.5
Three	276	315	39	295.5
Four	302	336	34	319
Five	291	343	52	317
Six	300	330	30	315

**Table 5:** The measured depth values after different numbers of repeated laser firings with the 4w_30k_300_re10 recipe.

Number of Repeated Laser Firings	Depth on the Left Edge (µm)	Depth on the Right Edge (µm)	Depth Difference (µm)	Depth Average (µm)
One	15.90	22.97	7.07	19.44
Two	37.10	45.94	8.84	41.52
Three	70.67	77.76	7.09	74.22
Four	72.46	70.67	1.79	71.57

**Table 6:** The measured depth values after different numbers of repeated laser firings with the 4w_30k_300_re20 recipe.

Number of Repeated Laser Firings	Depth on the Left Edge (µm)	Depth on the Right Edge (µm)	Depth Difference (µm)	Depth Average (µm)
One	24.73	30.09	5.36	27.41
Two	65.37	93.64	28.27	79.51
Three	132.51	123.69	8.82	128.10
Four	153.71	166.08	12.37	159.90

**Table 7:** The measured depth values after different numbers of repeated laser firings with the 4w_30k_100_re10 recipe.

Number of Repeated Laser Firings	Depth on the Left Edge (µm)	Depth on the Right Edge (µm)	Depth Difference (µm)	Depth Average (µm)
One	65.39	61.84	3.55	63.62
Two	98.96	104.25	5.29	101.61
Three	120.14	134.28	14.14	127.21
Four	162.54	178.45	15.91	170.50

**Table 8:** Summary of parameters before laser trimming and after laser trimming (measurements and simulation results).

	f_L_ (GHz)	f_H_ (GHz)	BW (MHz)	Highest S_11_ Value in Passband (dB)	S_21_ > −3 dB in Passband?	Image Rejection < −60 dB at ~5.5 GHz
Measured data (before)	4.991	5.178	187	−5.76	Yes (Bad S11)	Yes
Measured data (after)	5.15	5.33	180	−11.77	Yes	Yes

**Table 9:** Comparisons between the conventional LTCC process and the proposed process.

	LTCC Process [7]	Proposed Process
Temperature of metallization process (°C)	900~1000	<50
Metal clogged in holes	Yes	No
Minimum metal line width (µm)	100	50
Relative position of lines (µm)	±100	±50
Mass production	Yes	Not yet

## Data Availability

The data presented in this study are available on request from the corresponding author. The data are not publicly available due to their association with an ongoing study.

## Appendix A

**Table A1:** The simulation results obtained with a fixed digging radius of 0.4 mm and various digging depths.

Digging Hole Depth (mm)	f_L_ (GHz)	f_H_ (GHz)	BW (MHz)	The Highest S_11_ Value in Passband (dB)	S_21_ < −3 dB in Passband?
0	4.984	5.132	148	−4.20	5.028–5.049 < −3 dB
0.05	5.008	5.176	168	−5.97	Yes (Bad S_11_)
0.1	5.043	5.256	213	−10.84	Yes
0.15	5.076	5.293	217	−10.43	Yes
0.2	5.131	5.281	150	−5.85	Yes (Bad S_11_)

**Table A2:** The simulation results obtained with a fixed digging radius of 0.5 mm and various digging depths.

Digging Hole Depth (mm)	f_L_ (GHz)	f_H_ (GHz)	BW (MHz)	The Highest S_11_ Value in Passband (dB)	S_21_ < −3 dB in Passband?
0	4.984	5.132	148	−4.20	5.028–5.049 < −3 dB
0.05	5.022	5.220	198	−6.66	Yes (Bad S_11_)
0.1	5.061	5.274	213	−13.05	Yes
0.15	5.104	5.315	211	−5.05	5.280–5.303< −3 dB
0.2	5.245	5.292	47	-----	Fail

**Table A3:** The simulation results obtained with a fixed digging depth of 0.1 mm and various digging radii.

Digging Hole Depth (mm)	f_L_ (GHz)	f_H_ (GHz)	BW (MHz)	The Highest S_11_ Value in Passband (dB)	S_21_ < −3 dB in Passband?
0.4	5.043	5.256	213	−10.84	Yes
0.425	5.045	5.260	215	−11.30	Yes
0.45	5.050	5.265	215	−11.94	Yes
0.475	5.056	5.270	214	−12.50	Yes
0.5	5.061	5.274	213	−13.05	Yes

**Table A4:** The simulation results obtained with a fixed digging depth of 0.15 mm and various digging radii.

Digging Hole Depth (mm)	f_L_ (GHz)	f_H_ (GHz)	BW (MHz)	The Highest S_11_ Value in Passband (dB)	S_21_ < −3 dB in Passband?
0.4	5.076	5.293	217	−10.43	Yes
0.425	5.087	5.301	214	−8.33	Yes (Bad S_11_)
0.45	5.093	5.306	213	−6.95	Yes (Bad S_11_)
0.475	5.097	5.311	214	−5.92	Yes (Bad S_11_)
0.5	5.104	5.315	211	−5.05	5.280–5.303 < −3 dB
